# Prognostic value of the lactate dehydrogenase to albumin ratio in cancer patients

**DOI:** 10.3389/fnut.2025.1610487

**Published:** 2025-07-07

**Authors:** Dongqi Chai, Tao Yang, Lilong Zhang, Yuanjian Hui, Jiarui Feng, Weixing Wang

**Affiliations:** ^1^Department of General Surgery, Renmin Hospital of Wuhan University, Wuhan, Hubei Province, China; ^2^Hubei Key Laboratory of Digestive System Disease, Wuhan, Hubei Province, China; ^3^General Surgery Laboratory, Renmin Hospital of Wuhan University, Wuhan, Hubei Province, China; ^4^Department of General Surgery, Taihe Hospital, Hubei University of Medicine, Shiyan, Hubei Province, China

**Keywords:** immune checkpoint inhibitors, cancer, lactate dehydrogenase to albumin ratio, prognosis, hepatocellular carcinoma

## Abstract

**Objective:**

This study aimed to explore the prognostic relevance of the lactate dehydrogenase-to-albumin ratio (LAR) in cancer patients.

**Methods:**

A comprehensive literature search was conducted across PubMed, EMBASE, and the Cochrane Library for studies published before March 15, 2025. The primary outcomes included pooled hazard ratios (HRs) and corresponding 95% confidence intervals (CIs) for overall survival (OS), progression-free survival (PFS), and recurrence-free survival (RFS). In addition, a retrospective cohort of 71 hepatocellular carcinoma (HCC) patients treated with immune checkpoint inhibitors at our institution was analyzed to assess the prognostic impact of baseline LAR on OS and PFS.

**Results:**

Eighteen studies comprising 8,335 patients were incorporated into the meta-analysis. Elevated LAR was consistently associated with poorer outcomes: OS (HR: 2.02, 95% CI: 1.74–2.34, *p* < 0.001), PFS (HR = 1.35, 95% CI: 1.14–1.61, *p* < 0.001), and RFS (HR = 1.97, 95% CI: 1.47–2.64, *p* < 0.001). Subgroup evaluations stratified by LAR thresholds, geographical regions, treatment regimens, and statistical models confirmed the robustness of these associations. In our institutional cohort, patients presenting with pretreatment higher LAR experienced significantly diminished OS (HR = 2.04, 95% CI: 1.19–3.57, *p* = 0.008) and PFS (HR = 1.89, 95% CI: 1.14–3.13, *p* = 0.01) compared with those having lower LAR levels.

**Conclusion:**

These findings underscore the prognostic value of pretreatment LAR in cancer patients. Integrating LAR into clinical decision-making may aid clinicians in enhancing risk stratification and personalizing treatment strategies.

## 1 Introduction

Despite major strides in cancer prevention, early detection, and therapeutic innovation over recent decades, malignancies persist as the second most common cause of mortality globally (1). While survival has markedly improved across numerous tumor types, the overall burden of cancer continues to escalate (2). Current projections indicate that cancer-related deaths, estimated at 10 million in 2022, may climb to 16.3 million by 2040, primarily driven by demographic aging and shifting environmental and lifestyle-related exposures (3). As therapeutic advancements prolong patient survival, the population of individuals living beyond a cancer diagnosis is anticipated to expand considerably (4). However, many of these survivors face long-term complications arising from both the malignancy and its associated treatments (5). As such, the medical community is increasingly focused on identifying robust biomarkers to refine prognostic evaluation. These tools could inform the intensification of care for patients at elevated risk of recurrence and, conversely, support therapeutic de-escalation in those with more favorable disease trajectories, thereby minimizing unnecessary treatment-related toxicity (6–8).

Currently, there is broad consensus that an individual's immune competence and systemic inflammatory milieu are critically linked to therapeutic responsiveness and cancer prognosis (9). Owing to its accessibility and minimally invasive nature, peripheral blood serves as a valuable source for assessing inflammation-related biomarkers that may influence clinical trajectories in oncology patients (10). In light of this, it becomes particularly valuable to identify relevant biochemical indices and evaluate their combined prognostic relevance in determining individualized outcomes (11).

A range of hematological and nutritional indices has been evaluated in the oncology setting, including the neutrophil-to-lymphocyte ratio (12), lymphocyte-to-monocyte ratio (11), prognostic nutritional index (8), and the controlling nutritional status score (13). Lactate dehydrogenase (LDH), a key enzyme in anaerobic glycolysis, has been implicated in tumorigenesis, modulation of the immune milieu, and malignant progression (14). Elevated LDH levels prior to treatment initiation have been consistently linked to unfavorable survival outcomes across malignancies (15). Likewise, serum albumin—reflecting the host's nutritional reserve—has demonstrated prognostic value in oncologic populations (14). Notably, the LDH-to-albumin ratio (LAR), which integrates metabolic inflammation and nutritional depletion, has not yet been comprehensively investigated through evidence-based frameworks in cancer research (16).

Although the LAR has shown promise as a convenient prognostic indicator for cancer patients, variations across existing studies—including differences in study design, participant characteristics, and sample sizes—have limited the generalizability of individual findings. To address this, we conducted a systematic review of the current literature to clarify the relationship between LAR and clinical outcomes in oncology populations.

## 2 Methods

### 2.1 Search strategy, inclusion criteria, and exclusion criteria for the meta-analysis

This systematic review was conducted in accordance with the PRISMA (Preferred Reporting Items for Systematic Reviews and Meta-Analyses) guidelines (17). A thorough literature search was carried out across three major databases—PubMed, Cochrane Library, and EMBASE—to capture all relevant publications available until 15 March 2025. The search strategy employed predefined keywords such as “Lactate Dehydrogenase-to-albumin Ratio” and “Lactic Dehydrogenase-albumin Ratio” to ensure coverage of all relevant topics. A comprehensive overview of the search methodology is available in Supplementary Material 1. In addition, the reference sections of eligible studies were manually examined to identify potentially overlooked articles. To ensure methodological rigor and minimize selection bias, two reviewers (CD and YT) independently conducted the screening process. In case of any dispute, it shall be adjudicated by the senior author (WW).

Eligible studies were selected based on the following inclusion criteria: (1) retrospective or prospective investigations assessing the relationship between LAR and survival outcomes, including progression-free survival (PFS), recurrence-free survival (RFS), and overall survival (OS); (2) classification of participants into high and low LAR groups; (3) provision of hazard ratios (HRs) and corresponding 95% confidence intervals (CIs) comparing these groups; and (4) availability of full-text articles published in English. Studies were excluded if they met any of the following: (1) duplicate records; (2) case series, abstracts, case reports, review articles, editorials, or guidelines. When multiple investigations included shared patient cohorts, preference was given to those reports that demonstrated methodological robustness and provided the most extensive dataset (18).

### 2.2 Data extraction and quality assessment for the meta-analysis

In the course of extracting data, we methodically collected key study attributes, including authorship, year of publication, study timeframe, study location, cancer classification, therapeutic interventions, cohort size, patient demographics (such as age and sex), and cut-off values for LAR. Multivariate models were the primary source for deriving HRs. When multivariate results were not available, univariate models or Kaplan-Meier estimates were used instead. NOS (Newcastle-Ottawa Scale) is a tool for assessing the risk of bias in non-randomized studies. It is mainly used to evaluate the quality of case-control studies and cohort studies (19). A score of 6 or above indicates high methodological quality.

### 2.3 Study cohort and data collection for the retrospective study

We also conducted a retrospective study using data from our center to analyze the association of baseline LAR with HCC outcomes. This study received approval from the institutional review board (2020WDRM0203). Given its retrospective design, the requirement for informed consent was waived. We evaluated patients diagnosed with hepatocellular carcinoma (HCC) who underwent immune checkpoint inhibitors (ICIs) therapy between 2020 and 2022. Therapeutic regimens consisted of anti-PD-1 or anti-PD-L1 agents. Eligibility required at least one measurable lesion in accordance with RECIST version 1.1 criteria. Individuals were excluded if they had previously received ICIs or lacked a baseline LDH and albumin.

Detailed clinical data were extracted from electronic medical records, including age, sex, Eastern Cooperative Oncology Group performance status (ECOG PS), hepatitis origin, presence of cirrhosis, Barcelona Clinic Liver Cancer (BCLC) stage, Child–Pugh classification, number of tumors, macrovascular invasion status, line of treatment, modified albumin-bilirubin (mALBI) score, alpha-fetoprotein concentration, serum albumin, and LDH levels. The LAR was computed using the formula: LAR = LDH (U/L) ÷ ALB (g/L). Tumor progression was evaluated using RECIST v1.1 guidelines. Follow-up CT scans were routinely scheduled at 1- to 2-month intervals following treatment initiation. PFS was defined as the time span from the first administration of immune checkpoint inhibitors to either radiological progression or death. OS was measured from treatment initiation to death from any cause.

### 2.4 Statistical methods

Categorical variables were presented as counts alongside corresponding percentages. For comparisons between groups, either Fisher's exact test or the chi-square test was employed, based on test assumptions. Continuous data were described using either the median with interquartile ranges or the mean with standard deviation, as appropriate. Group differences in continuous variables were evaluated using independent samples *t*-tests. The Cox proportional-hazards model and the Kaplan-Meier method were used to assess survival curves across different groups.

Meta-analysis was conducted using Stata version 18.0, with results visually represented through forest plots. To assess inter-study variability, both Cochran's Q test and *I*^2^ index were employed, with heterogeneity deemed significant when the *I*^2^ statistic surpassed 25% (20). In cases of marked heterogeneity, analyses were conducted using the DerSimonian–Laird random-effects model; otherwise, a fixed-effects approach based on the Inverse Variance method was adopted. Potential publication bias was examined through both Begg's and Egger's tests (7). The robustness of the results was further confirmed by sensitivity testing, wherein each study was systematically excluded in turn to evaluate its individual impact on pooled estimates (21). Additional subgroup analyses were undertaken, stratifying the data by LAR cutoff levels, Cox model, treatments, and Country. Statistical significance was determined using a two-sided *p*-value threshold of <0.05.

## 3 Results

### 3.1 Search results and study characteristics

**Figure 1:**
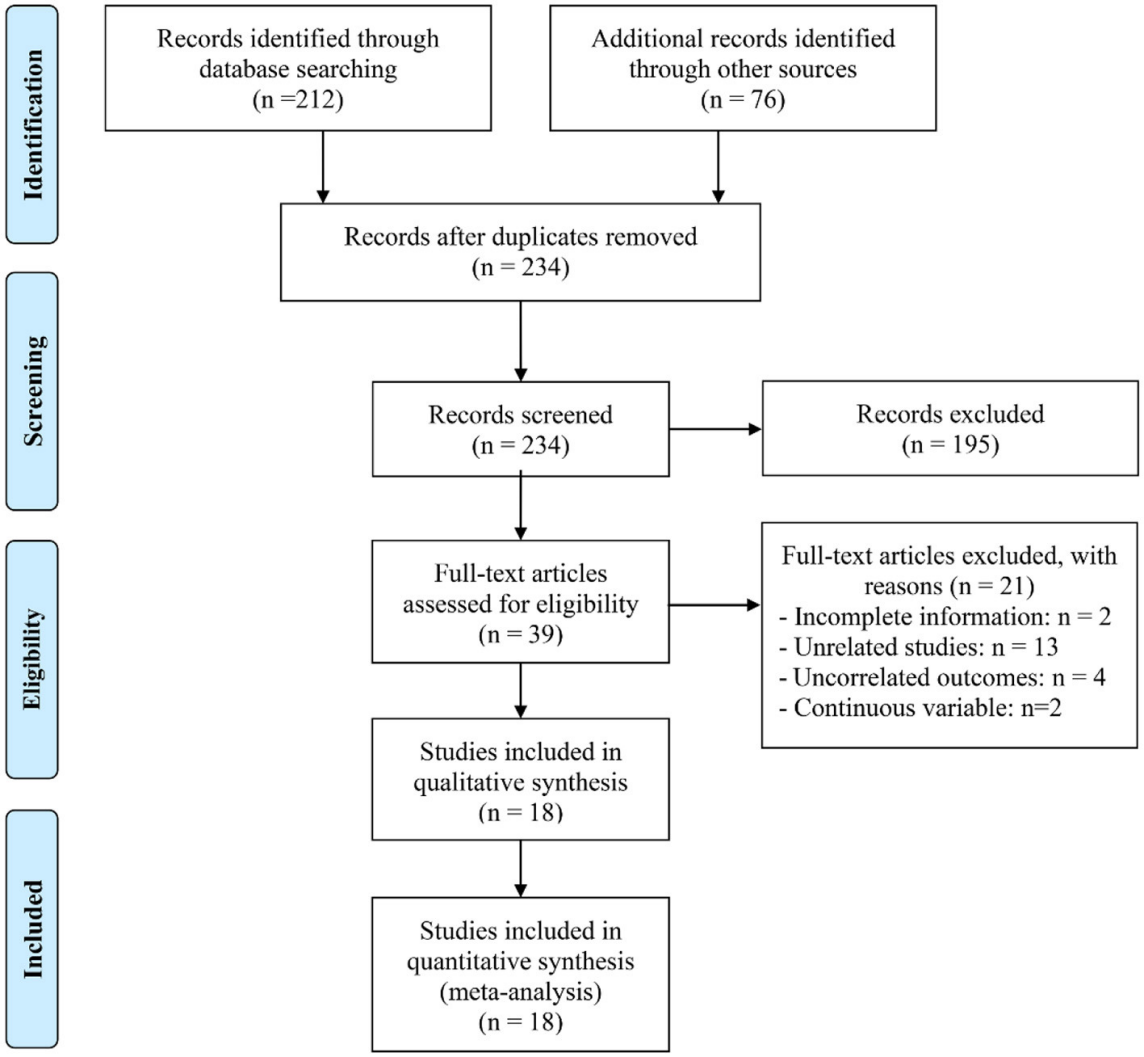
The flow diagram of identifying eligible studies.

The initial database query, complemented by manual reference list screening, identified 288 records deemed potentially relevant. After eliminating 54 duplicates, 195 entries were removed based on title and abstract evaluation due to failure to meet the inclusion criteria. A detailed review of the remaining 39 full-text papers led to the exclusion of 21 that did not fulfill the prespecified eligibility requirements. Ultimately, 18 article were retained for the final meta-analysis (22–39) (Figure 1).

Table 1 provides an overview of the key characteristics of the included studies. In total, 8,335 individuals were enrolled, with sample sizes ranging from 44 to 3,868 per study. Of the 18 studies, 12 were conducted in China, five in Turkey, and one in Mexico. Four studies involved patients with non-small cell lung cancer, three with colorectal cancer, two with esophageal carcinoma, two with gastric cancer, two with nasopharyngeal carcinoma, and two with breast cancer. Patients in 11 studies underwent surgery, while those in three studies received chemoradiotherapy, and three others received ICIs. All studies employed a retrospective design. Based on the NOS, quality scores ranged from 6 to 8, indicating a low risk of bias (Table 1 and Supplementary Table S2).

**Table 1:** Main characteristics of the studies included.

**Study**	**Study period**	**Country**	**Sample size**	**Age**	**Gender (male/female)**	**Treatment**	**Cancer type**	**Cut-point**	**NOS**
Shu et al. 2023	01/2011–01/2020	China	3,868	62.9^a^	2,279/1,589	Surgery	CRC	12.3	8
Luo et al. 2025	05/2019–03/2023	China	210	58.6 ± 10.6	166/44	ICIs	NSCLC	5.0	7
Shiratori et al. 2023	09/2008–03/2020	China	236	66 (41–83)^b^	193/43	Surgery	Esophageal Carcinoma	6.2	8
Peng et al. 2021	01/2010–12/2015	China	1,162	45.2 ± 10.8	860/302	Chemoradiotherapy	Nasopharyngeal Carcinoma	4.0	8
Çaglar et al. 2023	2016–2020	Turkey	91	63.4 ± 12.1	63/28	Surgery	Gastric cancer	5.5	7
Xie et al. 2022	06/2012–12/2015	China	126	66 (19–89)^c^	66/60	Surgery	CRC	4.9	7
He et al. 2023	04/2017–09/2018	China	134	51 (27–77)^b^	134	Surgery	Breast Cancer	3.4	7
Aday et al. 2020 (G)	06/2013–06/2019	Turkey	81	60.2 ± 13.8	55/26	Surgery	Gastric cancer	5.5	6
Aday et al. 2020 (C)	01/2013–06/2019	Turkey	295	55.8 ± 14.1	178/117	Surgery	CRC	5.3	7
Reyes-Pérez et al. 2023	01/2015–01/2022	Mexico	44	34 (27–43)^c^	26/18	Chemoradiotherapy	Hodgkin's lymphoma	12.5	6
Feng et al. 2019	01/2007–12/2010	China	346	147/199^d^	270/76	Surgery	Esophageal Carcinoma	5.5	8
Wang et al. 2024	01/2018–12/2019	China	190	122/68	108/82	Surgery	Oral Cancer	3.8	8
Arici et al. 2024	01/2015–06/2023	Turkey	304	50 (23–78)^b^	0/304	Surgery	Breast Cancer	4.7	7
Zhao et al. 2023	2011–2019	China	400	48 (40–55)^c^	287/113	Chemoradiotherapy	Nasopharyngeal Carcinoma	4.5	7
Xu et al. 2023	12/2010–05/2020	China	595	65 (58–72)^c^	507/88	Surgery	Bladder Cancer	3.8	7
Wu et al. 2023	01/2017–10/2022	China	160	67/65^e^	73/87	EGFR-TKIs	NSCLC	5.0	8
Menekse et al. 2023	2019–2023	Turkey	144	61 (35–78)^b^	96/48	ICIs	NSCLC	6.3	7
Lei et al. 2024 (T)	09/2019–06/2023	China	108	82/26^d^	83/25	ICIs	NSCLC	4.3	6

a mean,

b median (range),

c median (IQR),

d >60/ <60,

e >65/ <65. ICIs, immune checkpoint inhibitors; NSCLC, non-small cell lung cancer; CRC, colorectal cancer; EGFR-TKIs, epidermal growth factor receptor-tyrosine kinase inhibitors.

### 3.2 Baseline dehydrogenase/albumin ratio and overall survival

**Figure 2:**
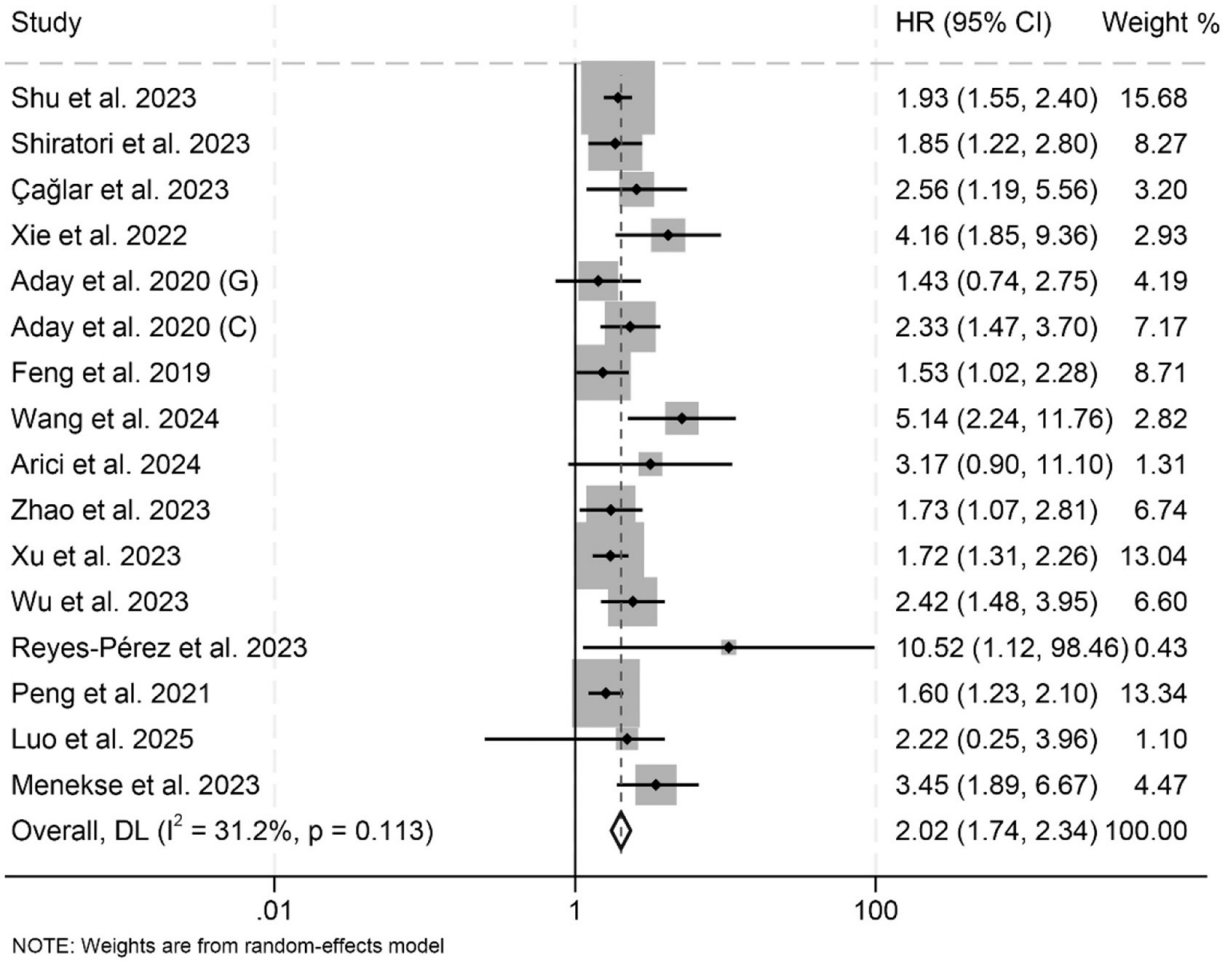
Forest plots depicting the association between the baseline lactate dehydrogenase/albumin ratio and overall survival in cancer patients. HR, hazard ratio; CI, confidence interval.

In this meta-analysis, a total of 16 eligible studies encompassing 8,253 patients were systematically examined to evaluate the prognostic significance of LAR on OS in cancers. The pooled HR demonstrated that high LAR was significantly correlated with inferior OS outcomes (HR: 2.02, 95% CI: 1.74–2.34, *p* < 0.001; Figure 2). Heterogeneity across studies was minimal, as evidenced by Cochran's Q and *I*^2^ metrics (*I*^2^ = 31.2%, *p* = 0.113), justifying the adoption of a fixed-effect model.

Subgroup analyses showed that both univariate and multivariate models consistently identified a significant association between elevated LAR and poorer OS (Table 2). This association remained robust across different treatment modalities and cancer types (Table 2). Additionally, variations in LAR cut-off values and differences in geographical location did not affect the strength or direction of the observed relationship between LAR and OS (Table 2).

**Table 2:** Subgroup analysis of the association between serum lactate dehydrogenase-to-albumin ratio and overall survival in cancer patients.

**Variable**	**Included studies**	**Test of association**			**Test of heterogeneity**		
		**HR**	**95%CI**	***p*-value**	**Modal**	***I* ^2^**	***p*-value**
**Cox model**							
Multivariate analysis	12	1.95	1.66–2.30	*p* < 0.001	F	32.8%	*p* = 0.128
Univariate analysis	4	2.33	1.66–3.26	*p* < 0.001	F	18.5%	*p* = 0.298
**Treatment**							
Surgery	10	2.01	1.68–2.41	*p* < 0.001	R	32.9%	*p* = 0.144
Chemoradiotherapy	3	1.71	1.23–2.40	*p* < 0.001	R	26.3%	*p* = 0.257
ICIs	2	3.20	1.80–5.67	*p* < 0.001	R	0	*p* = 0.570
**Cancer types**							
CRC	3	2.27	1.63–3.16	*p* < 0.001	R	42.8%	*p* = 0.174
EC	2	1.68	1.26–2.23	*p* < 0.001	R	0	*p* = 0.515
GC	2	1.85	1.04–3.26	*p* = 0.035	R	22.3%	*p* = 0.257
NSCLC	3	2.72	1.88–3.95	*p* < 0.001	R	0	*p* = 0.657
Other	6	2.01	1.48–2.74	*p* < 0.001	R	51.0%	*p* = 0.070
**Cut-off**							
> 6	4	2.19	1.59–3.03	*p* < 0.001	R	42.2%	*p* = 0.159
5–6	6	1.95	1.56–2.44	*p* < 0.001	R	0	*p* = 0.530
<5	6	2.14	1.56–2.93	*p* < 0.001	R	57.8%	*p* = 0.037
**Country**							
China	10	1.91	1.63–2.24	*p* < 0.001	R	32.8%	*p* = 0.145
Turkey	5	2.37	1.77–3.17	*p* < 0.001	R	0	*p* = 0.420

HR, hazard ratio; CL, confidence interval; R, random-effect model; F, fixed-effect model; ICIs, immune checkpoint inhibitors; EGFR-TKIs, epidermal growth factor receptor-tyrosine kinase inhibitors; CRC, colorectal cancer; EC, esophageal carcinoma; NSCLC, non-small cell lung cancer; GC, gastric cancer.

**Figure 3:**
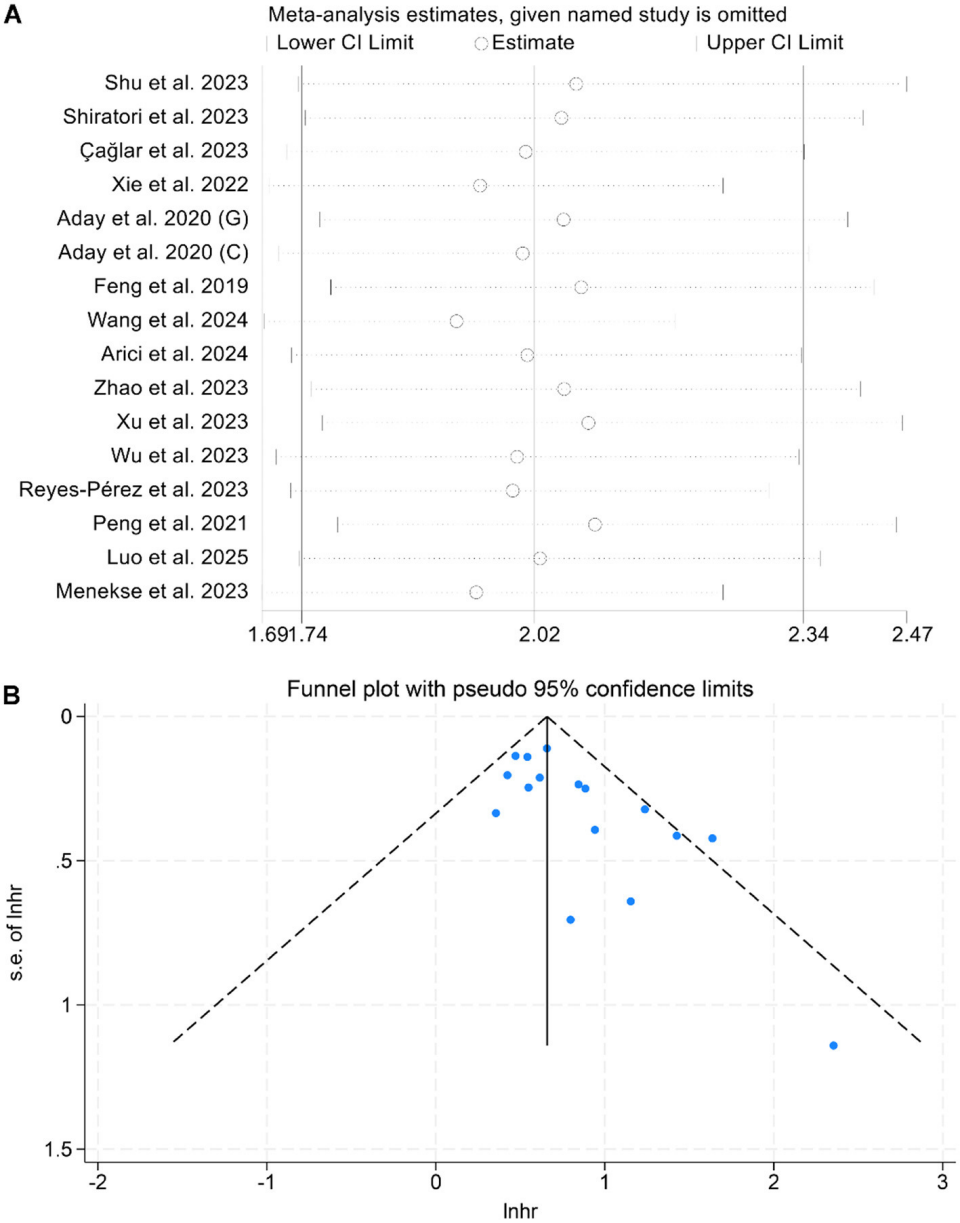
Sensitivity analysis of the association between baseline lactate dehydrogenase/albumin ratio and overall survival in cancer patients **(A)**. Funnel plots of the relationship between lactate dehydrogenase/albumin ratio and overall survival in cancer patients **(B)**. HR, hazard ratio; CI, confidence interval.

A sensitivity analysis, performed by sequentially omitting each study, confirmed that the combined HRs for OS remained stable and reliable (Figure 3A). Additionally, Begg's and Egger's tests detected no significant publication bias for OS (Begg: *p* = 0.142; Egger: *p* = 0.212). However, the funnel plot appeared to be asymmetrical (Figure 3B). Therefore, we conducted a trim-and-fill analysis to evaluate the potential impact of publication bias on the results. The analysis revealed that the overall conclusions remained unchanged after adjustment, indicating that potential publication bias did not materially affect our findings.

### 3.3 Baseline dehydrogenase/albumin ratio and progression-free survival

**Figure 4:**
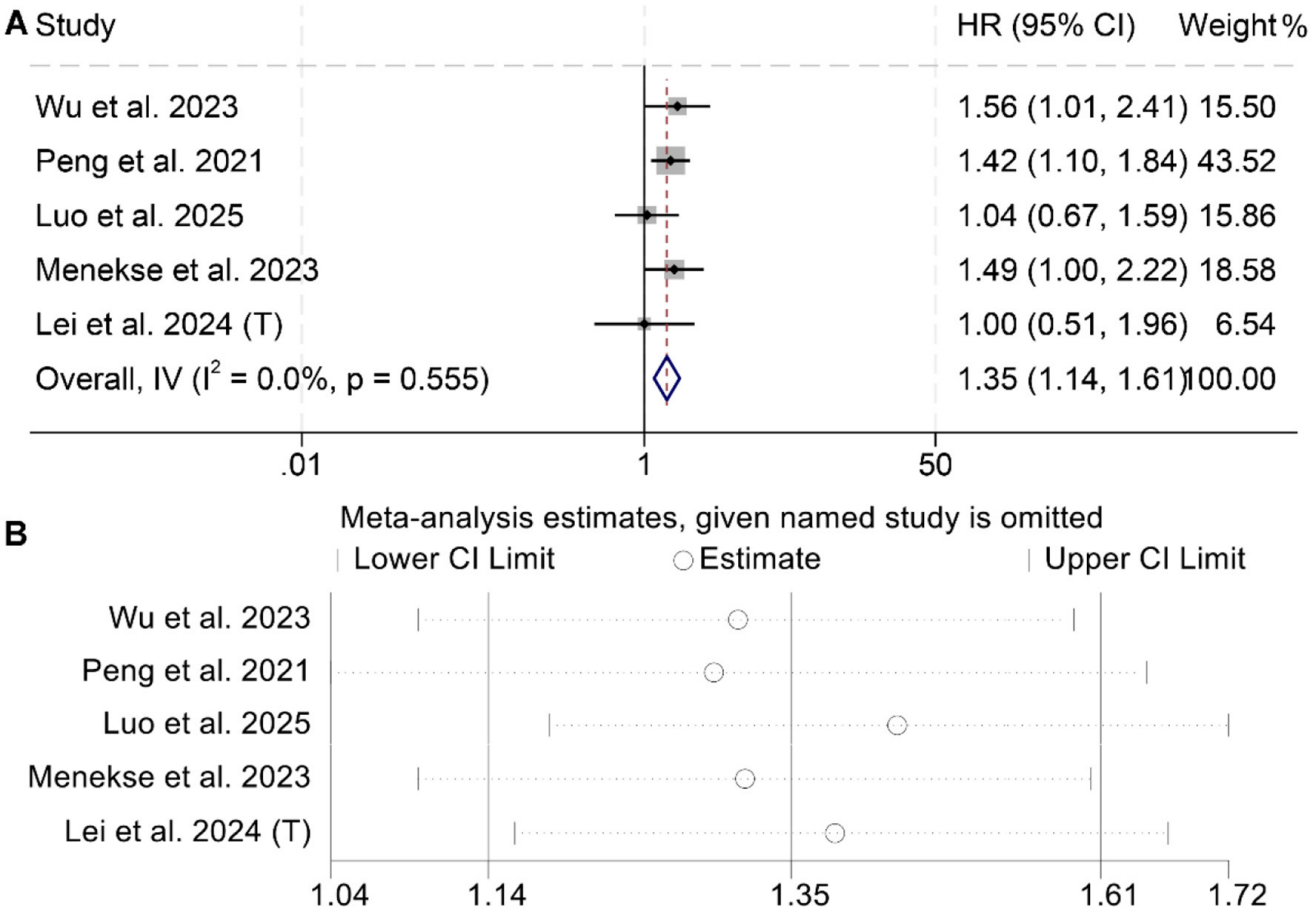
Forest plots depicting the association between the baseline lactate dehydrogenase/albumin ratio and progression-free survival in cancer patients **(A)**. Sensitivity analysis of the association between baseline lactate dehydrogenase/albumin ratio and progression-free survival in cancer patients **(B)**. HR, hazard ratio; CI, confidence interval.

A total of five studies, including 1,725 cancer patients, were analyzed to assess the association between LAR and PFS. Three studies reported a significant negative association between elevated LAR and OS, while two found no statistically significant relationship. The meta-analysis demonstrated that higher LAR was significantly associated with poorer PFS (*I*^2^ = 0, *p* = 0.555; HR = 1.35, 95% CI: 1.14–1.61, *p* < 0.001; Figure 4A). To test the stability of this finding, a sensitivity analysis was conducted by sequentially excluding each study, which confirmed that the pooled HR remained stable (Figure 4B). Additionally, publication bias was evaluated using Begg's and Egger's tests, both of which showed no significant evidence of bias (Begg's test: *p* = 1.000; Egger's test: *p* = 0.368).

### 3.4 Baseline dehydrogenase/albumin ratio and recurrence-free survival

**Figure 5:**
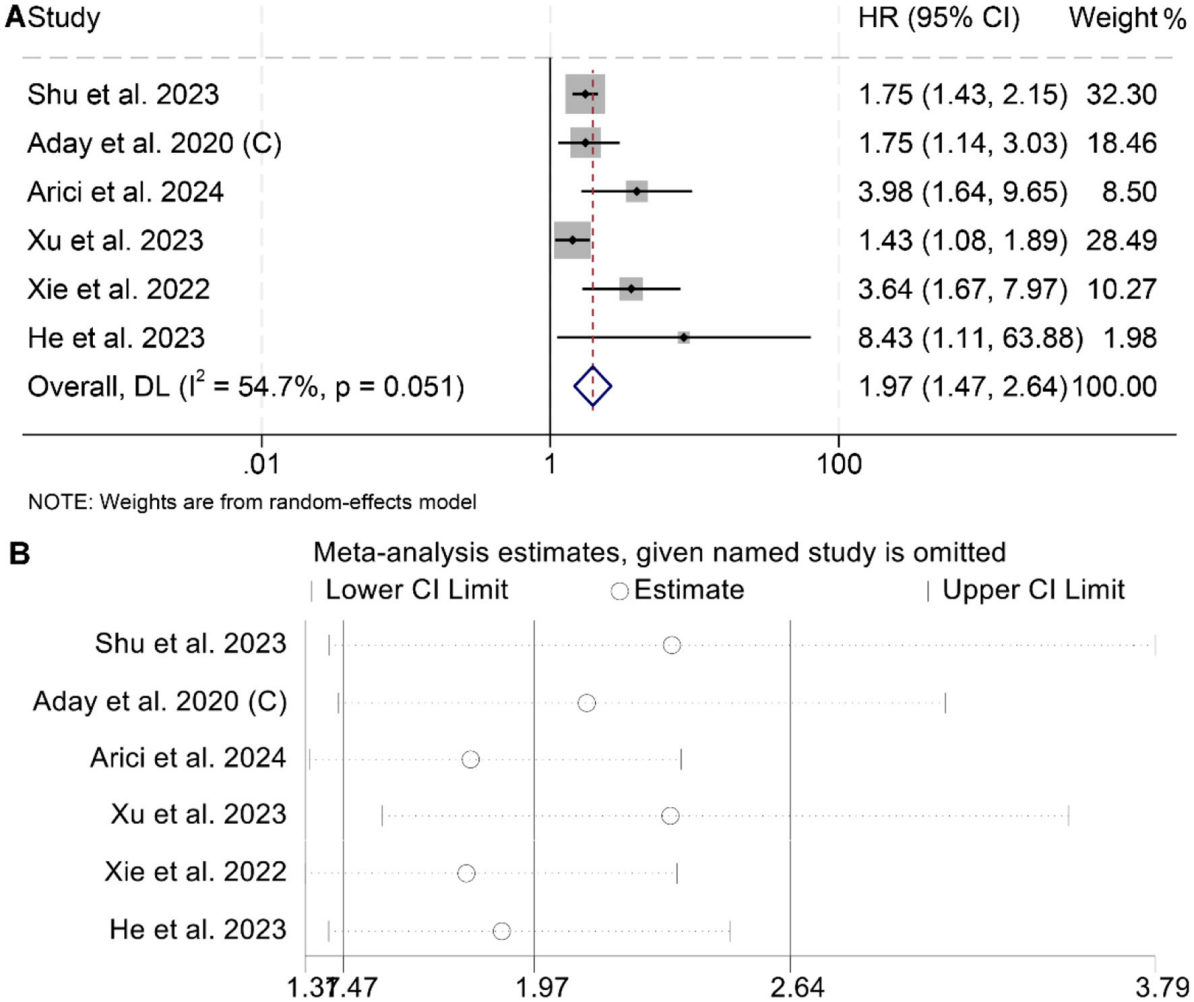
Forest plots depicting the association between the serum lactate dehydrogenase/albumin ratio and recurrence-free survival in cancer patients **(A)**. Sensitivity analysis of the association between lactate dehydrogenase/albumin ratio and recurrence-free survival **(B)**. HR, hazard ratio; CI, confidence interval.

The meta-analysis of six studies revealed that individuals with elevated baseline LAR had significantly poorer RFS compared to those with lower levels (HR = 1.97, 95% CI: 1.47–2.64, *p* < 0.001; Figure 5A). Substantial heterogeneity was observed among the studies (*I*^2^ = 54.7%, *p* = 0.051), warranting the use of a random-effects model. The robustness of the pooled effect estimate was confirmed through sensitivity analyses, which involved sequentially excluding each study and produced consistent results (Figure 5B). Additionally, Begg's and Egger's tests indicated no significant publication bias for RFS (Begg's test: *p* = 0.103; Egger's test: *p* = 0.260). Subgroup analysis confirmed that the above conclusion held true in all subgroups (Table 3).

**Table 3:** Subgroup analysis of the association between serum lactate dehydrogenase-to-albumin ratio and recurrence-free survival in cancer patients.

**Variable**	**Included studies**	**Test of association**			**Test of heterogeneity**		
		**HR**	**95%CI**	***p*-value**	**Modal**	**I^2^**	***p*-value**
**Cox model**							
Multivariate analysis	5	2.11	1.46–3.05	*p* < 0.001	R	63.8%	*p* = 0.026
Univariate analysis	1	1.75	1.07–2.87	*p* = 0.025	-	-	*-*
**Cut-off**							
> 5	2	1.75	1.45–2.11	*p* < 0.001	R	0	*p* = 0.993
<5	4	2.88	1.35–6.14	*p* = 0.006	R	72.8%	*p* = 0.012
**Country**							
China	4	1.88	1.33–2.66	*p* < 0.001	R	60.7%	*p* = 0.054
Turkey	2	2.42	1.11–5.31	*p* = 0.027	R	60.2%	*p* = 0.113

HR, hazard ratio; CL, confidence interval; R, random-effect model.

### 3.5 Baseline dehydrogenase/albumin ratio and prognosis in our cohort

Given the lack of existing studies on the relationship between LAR and prognosis in HCC patients, we analyzed data from our center to further contribute to the current understanding of LAR as a prognostic marker in cancer.

The demographic and clinical characteristics of the 71 HCC patients in our cohort are summarized in Supplementary Table 1. The median age was 62.4 years, ranging from 40.2 to 82.6 years. The majority of participants were male (59.15%, *n* = 42). In terms of functional status, 63.38% (*n* = 45) had an ECOG PS of 0, while 36.62% (*n* = 26) had a score of 1. Chronic viral hepatitis was present in 76.06% (*n* = 54) of patients, and hepatic cirrhosis was diagnosed in 63.38% (*n* = 45). According to the BCLC staging system, 7.04% (*n* = 5) were classified as early stage, 42.25% (*n* = 30) as intermediate stage, and 50.71% (*n* = 36) as advanced stage.

**Figure 6:**
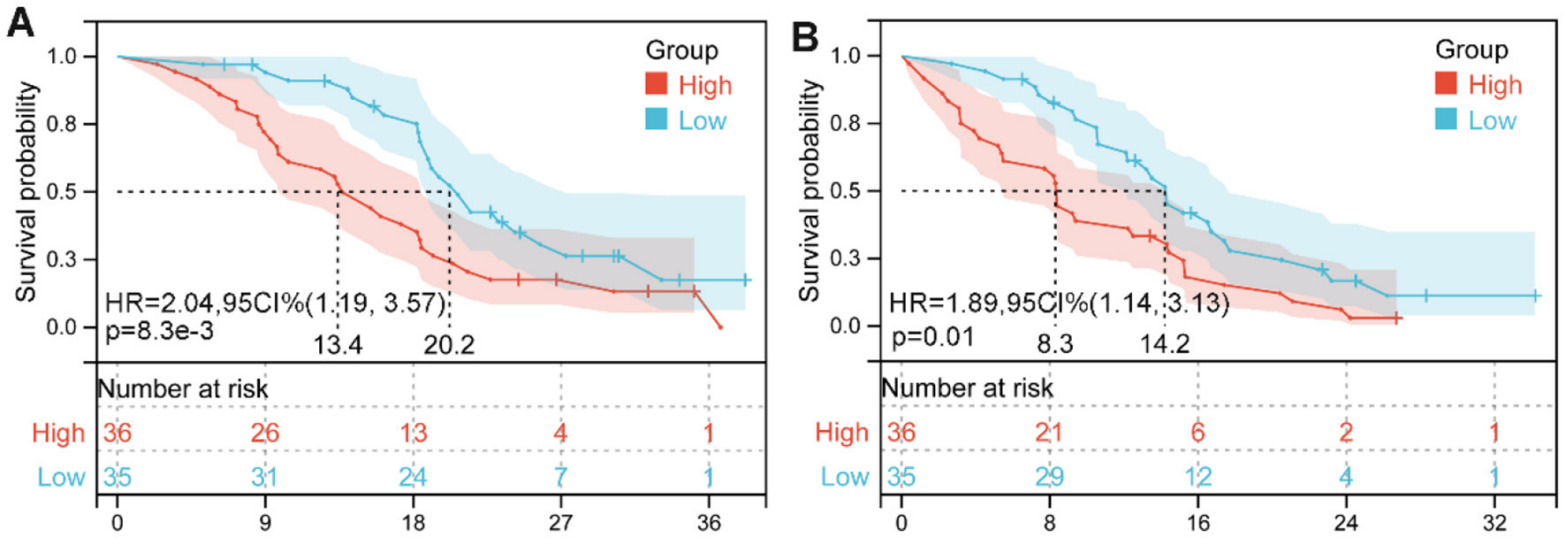
Kaplan–Meier survival estimates for overall survival **(A)** and progression-free survival **(B)** are presented, stratified by baseline lactate dehydrogenase-to-albumin ratio (LAR) levels in our cohorts. HR, hazard ratio; CI, confidence interval.

We divided the cohort into two groups based on the cutoff value for the median pretreatment LAR. Survival curves revealed significantly shorter OS (HR: 2.04, 95% CI: 1.19–3.57, *p* = 0.008, Figure 6A) and PFS (HR: 1.89, 95% CI: 1.14–3.13, *p* = 0.01; Figure 6B) in HCC patients with high LAR compared to those with low LAR.

## 4 Discussion

The LAR is a low-cost and easily obtainable biomarker derived from routine laboratory parameters. In the present analysis, elevated LAR levels were significantly associated with poorer survival outcomes in individuals with malignancies. Furthermore, subgroup analyses consistently supported the prognostic significance of LAR across various stratifications, including Cox regression models, treatment modalities, geographic regions, and LAR cut-off values.

LDH is a metabolic enzyme that catalyzes the interconversion of lactate and pyruvate within the cellular cytoplasm. In malignant cells, LDH plays a particularly critical role due to their preferential reliance on glycolysis for energy production—a metabolic shift known as the “Warburg effect” (40, 41). Instead of depending on mitochondrial oxidative phosphorylation, these cells primarily generate ATP through aerobic glycolysis, during which LDH facilitates lactate accumulation (42). This metabolic reprogramming leads to elevated lactate concentrations, resulting in acidification of the tumor microenvironment and promoting both cancer cell survival and invasiveness (43). Beyond its metabolic function, LDH also contributes to maintaining pH balance within the tumor niche, thereby supporting tumor growth and metastatic potential (44, 45). Numerous studies have shown that elevated circulating LDH levels are significantly associated with poor prognosis in various malignancies, including melanoma, prostate cancer, and renal cell carcinoma (46). These findings highlight the central role of LDH in the pathophysiology of solid tumors.

Albumin serves as an essential plasma protein involved in multiple physiological processes, particularly in maintaining oncotic pressure and facilitating the transport of vital substances such as hormones, fatty acids, and trace elements—functions that collectively uphold nutritional balance in the human body (47, 48). Beyond its nutritional role, albumin has emerged as a valuable prognostic marker in oncology (49). A diminished serum albumin concentration, termed hypoalbuminemia, has been consistently linked to adverse outcomes in a range of malignancies, highlighting its relevance in tumor biology (50). Within the immune milieu, albumin contributes both energy and nutrients to immune cells, thereby modulating their functional performance. Moreover, its antioxidant activity, anti-inflammatory effects, and role in mediating cytokine distribution and lymphocyte responsiveness further underscore its immunological significance (51). An in-depth understanding of albumin's diverse biological functions may offer new perspectives for optimizing therapeutic strategies and clinical decision-making in cancer care.

By incorporating both LDH, a surrogate marker of tumor metabolic activity, and albumin, which reflects systemic nutritional status, the LAR provides a comprehensive snapshot of the patient's physiological and oncological state. This integrated parameter may offer superior prognostic value compared to LDH or albumin when considered individually. Accumulating evidence supports LAR as an emerging prognostic biomarker across a wide range of malignancies. Our findings highlight the clinical relevance of incorporating LAR into standard pretreatment assessments, potentially enhancing therapeutic planning and patient stratification.

The findings of this meta-analysis underscore the potential utility of the lactate dehydrogenase-to-albumin ratio (LAR) as a simple, cost-effective, and readily available prognostic biomarker across various cancers. Clinically, LAR may assist physicians in risk stratification, treatment planning, and follow-up scheduling by identifying patients with a poorer prognosis who may benefit from more aggressive or tailored therapeutic approaches. From a public health perspective, the use of routinely measured laboratory parameters to predict cancer outcomes could be particularly valuable in low-resource settings where access to advanced molecular testing is limited.

Nonetheless, this meta-analysis is subject to several limitations. Primarily, the reliance on retrospective cohort studies may compromise the robustness of the pooled estimates. Additionally, as the majority of the data originate from cohorts based in China and Turkey, the extrapolation of these results to other ethnic or geographic populations warrants caution. Furthermore, inconsistency in LAR threshold definitions among the included studies introduces an additional layer of heterogeneity. Although LAR shows promise as a prognostic biomarker, its advantages over established factors such as tumor stage and pathological subtype remain unclear, limiting its immediate clinical applicability. Finally, all the included studies seemed to be single-arm cohorts and lacked comparison groups, which greatly limited causal inference. To address these concerns, future investigations should aim to validate these findings through well-designed, prospective, multinational trials, thereby strengthening the generalizability of LAR's prognostic utility in cancers.

## 5 Conclusion

These findings underscore the prognostic value of pretreatment LAR in cancer patients. Integrating LAR into clinical decision-making may aid clinicians in enhancing risk stratification and personalizing treatment strategies.

## Data Availability

The original contributions presented in the study are included in the article/Supplementary material, further inquiries can be directed to the corresponding authors.
