# Supplementary material for: Prognostic value of the lactate dehydrogenase to albumin ratio in cancer patients
Source: Front Nutr. 2025 Jul 7;12:1610487. doi: 10.3389/fnut.2025.1610487 (PMC12277156; doi:10.3389/fnut.2025.1610487)
Supplement: Supplementary file 1 [file Table_1.docx]

Supplementary material 1. Detailed article search strategy

((((Lactic Dehydrogenase-to-albumin Ratio[Title/Abstract]) OR (Lactic Dehydrogenase to albumin Ratio[Title/Abstract])) OR (Lactic Dehydrogenase-albumin Ratio[Title/Abstract])) OR (Lactic Dehydrogenase/albumin Ratio[Title/Abstract])) OR ((((Lactate Dehydrogenase-to-albumin Ratio[Title/Abstract]) OR (Lactate Dehydrogenase to albumin Ratio[Title/Abstract])) OR (Lactate Dehydrogenase-albumin Ratio[Title/Abstract])) OR (Lactate Dehydrogenase/albumin Ratio[Title/Abstract]))

PubMed: n=62

EMBASE: n=126

Cochrane Library: n=24

| Supplementary Table 1. Patient characteristics | |
| --- | --- |
|  | Overall (n=71) |
| Age | 62.4 (40.2-82.6) |
| Males | 42 (59.15%) |
| ECOG PS |  |
| 0 | 45 (63.38%) |
| 1 | 26 (36.62%) |
| Etiology |  |
| Viral | 54 (76.06%) |
| Other | 17 (23.94%) |
| Liver cirrhosis |  |
| Yes | 45 (63.38%) |
| No | 26 (36.62%) |
| BCLC stage |  |
| Early | 5 (7.04%) |
| Intermediate | 30 (42.25%) |
| Advanced | 36 (50.71%) |
| Child-Pugh class |  |
| A | 57 (80.28%) |
| B | 14 (9.72%) |
| Tumor number |  |
| < 3 | 53 (74.65%) |
| ≥ 3 | 18 (25.35%) |
| Macrovascular invasion |  |
| Yes | 21 (29.58%) |
| No | 50 (70.42%) |
| mALBI grade |  |
| 1 | 32 (45.07%) |
| 2 | 39 (54.93%) |
| AFP (ng/mL) |  |
| ≥ 400 | 40 (56.34%) |
| < 400 | 31 (43.66%) |
| Data shown are means with range or numbers with percentage.  ECOG PS, Eastern Cooperative Oncology Group performance status; BCLC, Barcelona Clinic Liver Cancer; AFP, α-fetoprotein; mALBI grade, modified albumin-bilirubin grade | |

| Supplementary Table 2. Newcastle-Ottawa Scale | | | | |
| --- | --- | --- | --- | --- |
|  | Selection | Comparability | Exposure | Quality scores |
| Shu et al. 2023 | 4 | 2 | 2 | 8 |
| Luo et al. 2025 | 3 | 1 | 3 | 7 |
| Shiratori et al. 2023 | 4 | 1 | 3 | 8 |
| Peng et al. 2021 | 4 | 2 | 2 | 8 |
| Çağlar et al. 2023 | 4 | 0 | 3 | 7 |
| Xie et al. 2022 | 3 | 1 | 3 | 7 |
| He et al. 2023 | 4 | 0 | 3 | 7 |
| Aday et al. 2020 (G) | 3 | 1 | 2 | 6 |
| Aday et al. 2020 (C) | 3 | 2 | 2 | 7 |
| Reyes-Pérez et al. 2023 | 3 | 0 | 3 | 6 |
| Feng et al. 2019 | 4 | 2 | 2 | 8 |
| Wang et al. 2024 | 4 | 1 | 3 | 8 |
| Arici et al. 2024 | 3 | 1 | 3 | 7 |
| Zhao et al. 2023 | 3 | 2 | 2 | 7 |
| Xu et al. 2023 | 4 | 1 | 2 | 7 |
| Wu et al. 2023 | 4 | 1 | 3 | 8 |
| Menekse et al. 2023 | 3 | 2 | 2 | 7 |
| Lei et al. 2024 (T) | 3 | 0 | 3 | 6 |
